# Viscoelastic Biomechanical Properties of the Conventional Aqueous Outflow Pathway Tissues in Healthy and Glaucoma Human Eyes

**DOI:** 10.3390/jcm11206049

**Published:** 2022-10-13

**Authors:** Alireza Karimi, Reza Razaghi, Steven Padilla, Seyed Mohammadali Rahmati, J. Crawford Downs, Ted S. Acott, Mary J. Kelley, Ruikang K. Wang, Murray Johnstone

**Affiliations:** 1 Department of Ophthalmology and Visual Sciences, University of Alabama at Birmingham, Birmingham, AL 35233, USA; 2 Department of Ophthalmology, University of Washington, Seattle, WA 98109, USA; 3 School of Biological Sciences, Georgia Institute of Technology, Atlanta, GA 30332, USA; 4 Departments of Ophthalmology and Biochemistry and Molecular Biology, Casey Eye Institute, Oregon Health & Science University, Portland, OR 97239, USA; 5 Departments of Ophthalmology and Integrative Biosciences, Casey Eye Institute, Oregon Health & Science University, Portland, OR 97239, USA; 6 Department of Bioengineering, University of Washington, Seattle, WA 98105, USA

**Keywords:** trabecular meshwork, juxtacanalicular tissue, Schlemm’s canal, viscoelastic material model, beam elements, inverse finite element method

## Abstract

Background: Although the tissues comprising the ocular conventional outflow pathway have shown strong viscoelastic mechanical response to aqueous humor pressure dynamics, the viscoelastic mechanical properties of the trabecular meshwork (TM), juxtacanalicular connective tissue (JCT), and Schlemm’s canal (SC) inner wall are largely unknown. Methods: A quadrant of the anterior segment from two human donor eyes at low- and high-flow (LF and HF) outflow regions was pressurized and imaged using optical coherence tomography (OCT). A finite element (FE) model of the TM, the adjacent JCT, and the SC inner wall was constructed and viscoelastic beam elements were distributed in the extracellular matrix (ECM) of the TM and JCT to represent anisotropic collagen. An inverse FE-optimization algorithm was used to calculate the viscoelastic properties of the ECM/beam elements such that the TM/JCT/SC model and OCT imaging data best matched over time. Results: The ECM of the glaucoma tissues showed significantly larger time-dependent shear moduli compared to the heathy tissues. Significantly larger shear moduli were also observed in the LF regions of both the healthy and glaucoma eyes compared to the HF regions. Conclusions: The outflow tissues in both glaucoma eyes and HF regions are stiffer and less able to respond to dynamic IOP.

## 1. Introduction

Intraocular pressure (IOP) requires maintaining a time-averaged balance between the aqueous humor production and drainage via the trabecular meshwork (TM), juxtacanalicular connective tissue (JCT), and inner wall endothelium of Schlemm’s canal (SC) of the conventional outflow pathway [1,2,3,4]. Dysregulation in the balance between the aqueous humor inflow and outflow can result in an IOP elevation that is associated with primary open-angle glaucoma (POAG) [5,6,7,8,9,10,11,12]. The TM beams consist of highly organized dense collagen fibers, while the JCT contains a loosely arrayed extracellular matrix (ECM) containing collagen fibrils. The aqueous humor drains to the SC lumen and eventually flows circumferentially to the collector channels leading to the aqueous and episcleral veins [13,14,15,16,17,18].

The ECM in the JCT has been believed to act as a passive filter providing physical resistance to the aqueous humor across the TM. Recently, it has been shown that the TM mechanical properties play a major role in the resultant outflow resistance across the outflow pathway [19,20,21,22,23]. A major regulatory site of aqueous humor drainage in the conventional outflow pathway resides within the ECM of the JCT and the immediate vicinity of the SC inner wall [24,25,26,27,28,29]. It is suggested that the TM acts as a biomechanical pump that generates pulsatile flow [14,30,31,32]. Several studies have also shown that the aqueous outflow from the SC is pulsatile and synchronized with IOP dynamic [14,30,33,34,35].

IOP transients unable to transfer pressure gradients directly from the anterior chamber to the SC due to the complex, labyrinthine structure of the TM. However, the pulsatile flow in the SC may occur because of the TM movement in response to the cardiac pulse [36]. Transient IOP fluctuations [12,32,37,38,39,40,41,42] also cause a dynamic mechanical environment in the outflow pathway that actively affects the geometry and cellular mechanotransduction of the outflow tissues [43,44,45]. Active outflow resistance regulation in the conventional outflow pathway results in a dynamic outflow pressure gradient across the outflow tissues [9,46,47] that significantly contributes to the IOP homeostasis [17,48,49]. The TM mechanically responds to IOP dynamic by geometrical alterations due to an active, two-way, fluid–structure interaction (FSI) coupling between the outflow tissues and aqueous humor [28,50]. Thus, TM/JCT/SC complex motion and its resulting mechanisms of aqueous outflow resistance [51,52] largely depend on the biomechanical properties of the outflow tissues [36,53,54,55,56,57]. The TM motion has shown a strong correlation with IOP fluctuation [51,52] with ocular pulse amplitude [51] and velocity. Li [36] perfused nonhuman primate eyes (*Macaca nemestrina*) at a constant ocular pulse amplitude and measured the TM motion using phase-sensitive optical coherence tomography (PhS-OCT). They found a significant correlation between the TM displacement and the IOP load rate. Vranka [58] also found different elastic moduli for the TM in physiologic and elevated perfusion pressures using atomic force microscopy (AFM). These studies [36,58] provide evidence that the mechanical response of the TM is load-rate dependent (viscoelastic). Vranka also showed that the outflow is segmental with low-flow (LF) and high-flow (HF) regions and there are biomechanical and protein level differences between LF and HF segmental regions of the TM [58].

To date, experimental [54,59,60] and numerical [60,61] TM biomechanical studies have all been limited to the isotropic elastic and hyperelastic mechanical properties of the TM, while it is known that soft biological tissues [62,63,64,65,66,67], especially the TM [36,58], are anisotropic and viscoelastic. In our prior study, we calculated the viscoelastic mechanical properties of the TM/JCT/SC complex with embedded elastic cable elements [68] using an inverse finite element (FE) method coupled with an optimization algorithm and high resolution optical coherence tomography (HR-OCT) imaging data [69]. We showed that the viscoelastic material model had an advantage in addressing the time-dependent mechanical response of the outflow tissues compared to the elastic material model. However, the TM, JCT, and SC inner wall in our prior study were modeled with the same mechanical properties, while their anatomic features and pressure-dependent responses indicate that they have different mechanical properties [58,59]. In addition, elastic cable elements were used to address the anisotropic mechanical properties of the outflow tissues imparted by collagen fibrils, but it has been shown that the mechanical properties of collagen fibrils are also time-dependent [70,71]. Viscoelasticity is the time-dependent anelastic behavior of material causing a delayed response to a stimulus, thus resulting in a loss of energy inside the material. Viscoelastic behavior normally occurs at different time scales [72]. A typical load case scenario in the outflow pathway due to dynamic IOP is most likely ≤ 1 sec [41]. Viscoelasticity also strongly influences the magnitude and frequency of the mechanical loads on cells resident in the tissue. The load establishes the internal strain and morphology that determine responses to the cells’ biomechanical signals from their environment [73,74]. Viscoelastic material models have included the rate of the IOP load in the bulk assessment of stresses and strains in the outflow tissues [75,76,77,78]. However, we are unaware of any studies that have reported the component viscoelastic properties of the TM, JCT, and SC inner wall.

Our study aimed to characterize the TM, JCT, and SC inner wall viscoelastic properties by using an inverse FE method to best match TM/JCT/SC and high-resolution OCT measurements over time. The results of this study may contribute to finding a correlation between the ex vivo and time-varying in vivo stimuli [69,79,80,81] that can be used to design and fabricate ex vivo 3D cultures. The study may establish a physiologically relevant environment to study IOP regulation mechanisms. An optimized environment may allow us a more effective development of new generations of glaucoma devices and medications focusing on IOP regulation.

## 2. Materials and Methods

### 2.1. Human Donor Eyes, Organ Culture Prep, HR-OCT Imaging, and Pressure Validation

Two healthy and two glaucoma human eyes (European descent), ages 54 and 89, were obtained within 72 h postmortem from the Oregon VisionGift eye bank (Portland, OR, USA), and anterior segments were perfused under organ culture conditions [79] to measure the outflow facility and identify LF and HF regions in the TM [61]. Eye tissue procurement followed the principles of the Declaration of Helsinki. The experimental descriptions are explained in our prior publication [69]. Details on the glaucoma or normal donors included age, race, gender, and postmortem time from death to stationary culture in Portland, but not detailed ophthalmologist’s records.

**Figure 1:**
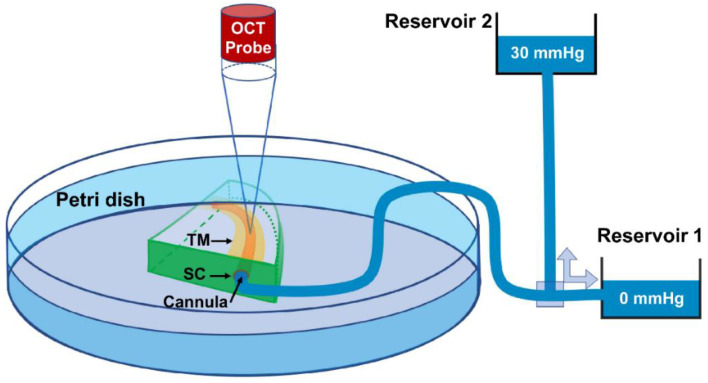
Schematic overview of experimental setup, including the HR-OCT system, two reservoirs used for controlling pressure in SC. HR-OCT B-scan images were acquired continuously through the TM, JCT and SC at 30 Hz, resulting in very high resolution images from which TM/JCT/SC complex motion can be determined in real time [61,69].

The anterior segment LF and HF wedges were dissected, placed interior up, and the SC cannulated as schematically shown in Figure 1 and explained in our prior publications [61,69]. Briefly, a quadrant of the anterior segment, including the cornea, limbal region with TM, SC, and ~5 mm of the sclera, was placed in a Petri dish and held in place with pins. The inner TM surface faced upward, and the entire quadrant was submerged in a saline bath (Figure 1) [61,69,80]. The saline bath also helped to eradicate surface interface motion artifacts due to experimentally induced dynamic TM motion [61]. A cannula was inserted into one end of SC with the help of a dissecting microscope and a micromanipulator device while the other end remained open. The micromanipulator ensured that the tip of the cannula maintained a tight contact with the interior walls of the SC lumen. The other end of the cannula was connected to a saline reservoir. The pressure in the SC lumen was controlled by changing the height of the reservoirs resulting in an oscillating pulsatile load boundary (Figure 1).

**Figure 2:**
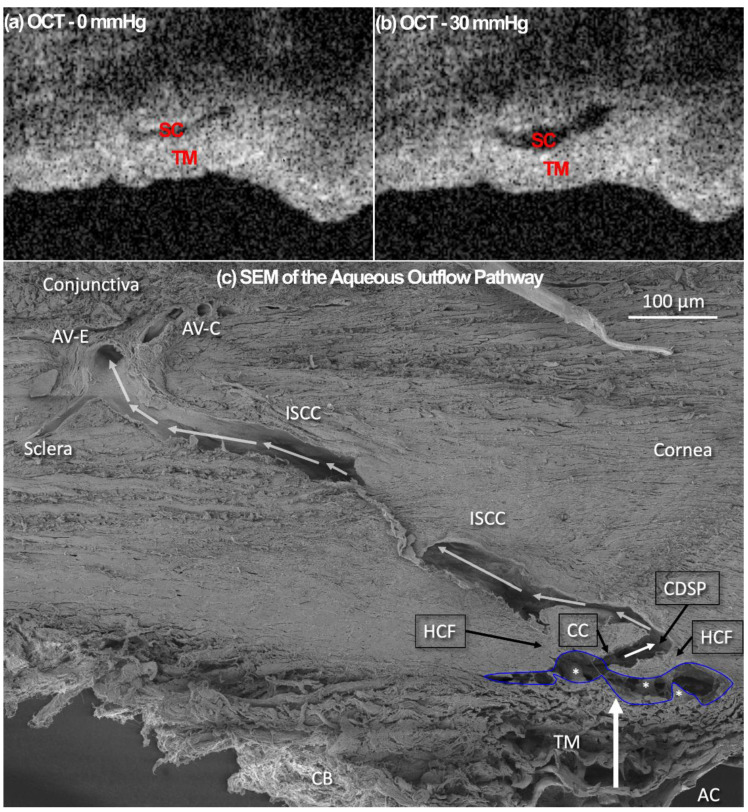
OCT images of the TM at the SC pressures of (**a**) 0 and (**b**) 30 mmHg. (**c**) Pathway of Aqueous Humor: Scanning electron microscopy of aqueous outflow pathway from the anterior chamber (AC) through the trabecular meshwork (TM) to Schlemm’s canal (outlined in blue). White arrows denote further aqueous passage through a circumferential deep intrascleral plexus (CDSP). Aqueous then flows through intrascleral collector channels (ISCC) to the episcleral and conjunctival aqueous veins, finally entering the systemic episcleral vein system. A collector channel (CC) entrance leads to a pathway between two hinged collagen flaps or leaflets. SC inlet valvelike structures (*) attach between SC inner wall and the hinged collagen flap (HCF). If the TM moves, the HCF must also move because each attaches to the SIV attachment structures. AV-E, aqueous veins in the episcleral; AV-C, aqueous veins in the conjunctiva; CB, ciliary body. *Macaca nemestrina* primate eye. From: Johnstone M. Intraocular pressure control through linked trabecular meshwork and collector channel motion. Glaucoma Research and Clinical Advances. Amsterdam: Kugler; 2016; pp. 41–85.

To capture a high-resolution dynamic movement of the TM/JCT/SC complex, the HR-OCT imaging probe was adjusted to face the TM (Figure 1). While the reservoir pressures were varied from 0 to 30 mmHg, a series of cross-sectional B-scans through the TM/JCT/SC complex were captured at multiple locations [61]. The spacing between two adjacent B-scans was ~10 μm. The OCT image of the TM/JCT/SC complex with the SC pressure of 0 and 30 mmHg, as well as a representative structural scanning electron microscopy (SEM) image from a limbal region, are shown in Figure 2.

**Figure 3:**
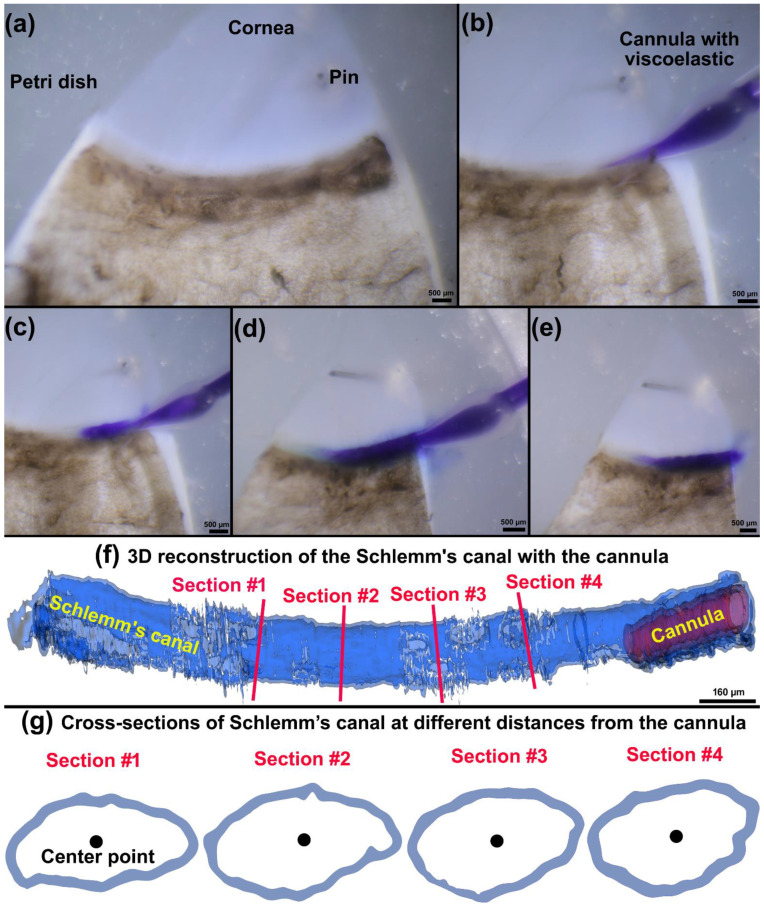
(**a**) A quadrant of the anterior segment was pinned in a Petri dish. (**b**) The SC was cannulated and (**c**,**d**) the viscoelastic was injected into the SC. (**e**) Once the viscoelastic is started to flow back from the SC lumen we removed the cannula. (**f**) The 3D reconstruction of the SC with the cannula after viscoelastic injection. (**g**) Cross-sections of the SC at different distances from the cannula. The distance between each cross section is ~200 µm.

The nominal pressure in the SC pressurization system oscillates from 0 to 30 mmHg, but the actual pressure in the SC may not be equivalent to that of the nominal pressure. In our pressurization system, only one end of the SC is cannulated while the other remains open, hence the pressure in SC is unknown. One may argue that significant aqueous will flow into collector channels or exit from the other end of the SC when we elevate the pressure from 0 to 30 mmHg. In addition, the cannula and the tubes may have their own flow resistance that would affect the pressure of the flow before leaving the cannula, as these flow resistance parameters have been fully calculated in our recent publication [61]. To address this concern, we cannulated and performed high-resolution OCT of SC to measure the resultant radial expansion in the SC at the reservoir pressure of 30 mmHg. The radial expansion was accomplished using a tissue wedge pinned to paraffin at the base of a Petri dish. Viscoelastic was injected into one end of the canal while the other remained open. The viscoelastic was Healon^®^ GV Pro, Johnson & Johnson Vision, New Brunswick, NJ, with a viscosity of 2000 Pa.s. The viscoelastic was gently and smoothly perfused into the SC using a specially designed cannula for SC pressurization. Injection steps are shown in Figure 3a–e. The viscoelastic was infused into the SC until it started to flow back around the cannula at the cannulation site (Figure 3d,e).

A series of cross-sectional B-scans through the TM/JCT/SC complex was captured within 800 µm from the cannula while maintaining a reservoir-dependent pressure of 30 mmHg. The SC and the cannula were segmented and reconstructed using a free, open-source, and multi-platform 3D Slicer image computing software [81] as shown in Figure 3f. Four different cross-sections were selected. A Matlab code was used to calculate the center point of the SC lumen and the average diameter of a best-fit circle to the SC inner lumen as described in our prior publications [68,82,83] and shown in Figure 3g. The goal was to see how far from the cannula the HR-OCT imaging results in a relatively similar radial expansion (≤5%) in the SC lumen. Our calculations assume that the SC lumen has homogenous material properties, so the mechanical properties of the SC walls throughout the lumen are the same.

### 2.2. Mesh-Free, Beam-in-Solid Coupling Algorithm

Our recently developed mesh-free, penalty-based, cable-in-solid coupling algorithm can simulate the anisotropic mechanical behavior of linear elastic or nonlinear hyperelastic collagenous tissues [68]. However, it is known that both the ECM and collagen fibrils are viscoelastic [84,85], so our previous approach cannot model the viscoelastic mechanical behavior of the ECM with embedded collagen fibrils. The Hughes-Liu beam element formulation [86] was therefore programmed based on the beam theory available in LS-DYNA (Ansys/LS-DYNA, Canonsburg, PA, USA) [87]. The approach permitted incorporating viscoelasticity into a beam formulation. The Hughes-Liu beam theory is incrementally objective, so rigid body rotations do not generate strains in the beams. Since the beam formulation is based on the degenerated hexahedral elements, it works well with both the 8-noded and 20-noded hexahedral elements. This element formulation includes finite transverse shear strains, so it computationally requires less effort to retain the strain component compared to those for the assumption of no transverse shear strain.

The Hughes-Liu beam element is based on a degenerated isoparametric 8-noded solid element proposed by Ahmad et al. [88]. The solid element isoparametric mapping of the biunit cube is as follows:

(1) $$x\left(\xi ,\eta ,\zeta \right)=N_{a}\left(\xi ,\eta ,\zeta \right)x_{a}$$

(2) $$N_{a}\left(\xi ,\eta ,\zeta \right)=\frac{\left(1+\xi_{a}\xi \right)\left(1+\eta_{a}\eta \right)\left(1+\zeta_{a}\zeta \right)}{8}$$

where x is an arbitrary point in the element, (ξ,η,ζ) are the parametric coordinates, $x_{a}$ are the global nodal coordinates of node a, and $N_{a}$ are the element shape functions evaluated as node *a*, e.g., $\left(\xi_{a},\eta_{a},\zeta_{a}\right)$ are (ξ,η,ζ) evaluated at node *a*.

**Figure 4:**
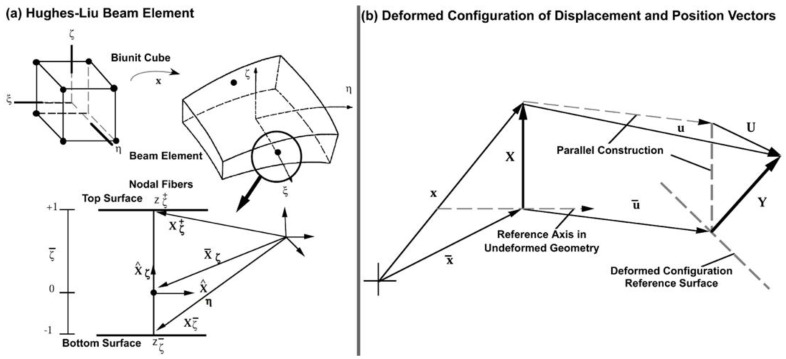
(**a**) Hughes-Liu Beam Element. (**b**) Deformed configuration of displacement and position vectors (LS-DYNA user manual: Open Access, Copyright is not required).

In the beam geometry, ξ determines the location along the axis of the beam and the coordinate pair (η,ζ) defines a point on the cross section. To degenerate the 8-noded brick geometry into the 2-noded beam geometry, the four nodes at ξ=−1 and at ξ=+1 are combined into a single node with three translational and three rotational degrees-of-freedom. Orthogonal and inextensible nodal beam elements are defined at each node for treating the rotational degrees of freedom. Schematics of the biunit cube and the beam element are shown in Figure 4a.

(3) $$x\left(\xi ,\eta ,\zeta \right)=\bar{x}\left(\xi \right)+X\left(\xi ,\eta ,\zeta \right)=\bar{x}\left(\xi \right)+X_{\zeta }\left(\xi ,\zeta \right)+X_{\eta }\left(\xi ,\eta \right)$$

where $\bar{x}$ denotes a position vector to a point on the reference axis of the beam, and $X_{\zeta }$ and $X_{\eta }$ are positions vectors at point $\bar{x}$ on the axis that define the beam element directions through that point.

(4) $$\bar{x}\left(\xi \right)=N_{a}\left(\xi \right){\bar{x}}_{a}$$

(5) $$X_{\eta }\left(\xi ,\eta \right)=N_{a}\left(\xi \right)X_{\eta a}\left(\eta \right)$$

(6) $$X_{\zeta }\left(\xi ,\zeta \right)=N_{a}\left(\xi \right)X_{\zeta a}\left(\zeta \right)$$

With this description, arbitrary points on the reference line $\bar{x}$ are interpolated by the one-dimensional shape function N(ξ) operating on the global position of the two beam nodes that define the reference axis, e.g., ${\bar{x}}_{a}$. Points off the reference axis are further interpolated by using a one-dimensional shape function along the beam element directions, e.g., $X_{\eta a}\left(\eta \right)$ and $X_{\zeta a}\left(\zeta \right)$ where [89]:

(7) $$X_{\eta a}\left(\eta \right)=z_{\eta }\left(\eta \right){\hat{X}}_{\eta a}$$

(8) $$z_{\eta }\left(\eta \right)=N_{+}\left(\eta \right)z_{\eta a}^{+}+N_{-}\left(\eta \right)z_{\eta a}^{-}$$

(9) $$N_{+}\left(\eta \right)=\frac{\left(1+\eta \right)}{2}$$

(10) $$N_{-}\left(\eta \right)=\frac{\left(1-\eta \right)}{2}$$

(11) $$X_{\zeta a}\left(\zeta \right)=z_{\zeta }\left(\zeta \right){\hat{X}}_{\zeta a}$$

(12) $$z_{\zeta }\left(\zeta \right)=N_{+}\left(\zeta \right)z_{\zeta a}^{+}+N_{-}\left(\zeta \right)z_{\zeta a}^{-}$$

(13) $$N_{+}\left(\zeta \right)=\frac{\left(1+\zeta \right)}{2}$$

(14) $$N_{-}\left(\zeta \right)=\frac{\left(1-\zeta \right)}{2}$$

where $z_{\zeta }\left(\zeta \right)$ and $z_{\eta }\left(\eta \right)$ are thickness functions.

Four position vectors are used in the Hughes-Liu beam formulation plus ξ, to locate the reference axis and define the initial beam element directions. Consider the two position vectors $x_{\zeta a}^{+}$ and $x_{\zeta a}^{-}$ located on the top and bottom surfaces, respectively, at node *a*. Then,

(15) $${\bar{x}}_{\zeta a}=\frac{1}{2}\left(1-\bar{\zeta }\right)x_{\zeta a}^{-}+\left(1+\bar{\zeta }\right)x_{\zeta a}^{+}$$

(16) $${\hat{X}}_{\zeta a}=\frac{\left(x_{\zeta a}^{+}-x_{\zeta a}^{-}\right)}{\left\|x_{\zeta a}^{+}-x_{\zeta a}^{-}\right\|}$$

(17) $$z_{\zeta a}^{+}=\frac{1}{2}\left(1-\bar{\zeta }\right).\|x_{\zeta a}^{+}-x_{\zeta a}^{-}\|$$

(18) $$z_{\zeta a}^{-}=-\frac{1}{2}\left(1+\bar{\zeta }\right).\|x_{\zeta a}^{+}-x_{\zeta a}^{-}\|$$

(19) $${\bar{x}}_{\eta a}=\frac{1}{2}\left(1-\bar{\zeta }\right)x_{\eta a}^{-}+\left(1+\bar{\zeta }\right)x_{\eta a}^{+}$$

(20) $${\hat{X}}_{\eta a}=\frac{\left(x_{\eta a}^{+}-x_{\eta a}^{-}\right)}{\left\|x_{\eta a}^{+}-x_{\eta a}^{-}\right\|}$$

(21) $$z_{\eta a}^{+}=\frac{1}{2}\left(1-\bar{\eta }\right).\|x_{\eta a}^{+}-x_{\eta a}^{-}\|$$

(22) $$z_{\eta a}^{+}=-\frac{1}{2}\left(1+\bar{\eta }\right).\|x_{\eta a}^{+}-x_{\eta a}^{-}\|$$

where ||.|| is the Euclidean norm. The reference surface may be located at the midsurface of the beam or offset at the outer surfaces. This capability is useful in several practical situations involving contact surfaces and connection of beam elements to solid ECM. The reference surfaces are located within the beam element by specifying the value of the parameters $\bar{\eta }$ and $\bar{\zeta }$ (Figure 4a). When these parameters take on the values −1 or +1, the reference axis is located on the outer surfaces of the beam. If they are set to zero, the reference axis is at the center [89].

(23) $$u\left(\xi ,\eta ,\zeta \right)=\bar{u}\left(\xi \right)+U\left(\xi ,\eta ,\zeta \right)=\bar{u}\left(\xi \right)+U_{\zeta }\left(\zeta ,\zeta \right)+U_{\eta }\left(\xi ,\eta \right)$$

(24) $$\bar{u}\left(\xi \right)=N_{a}\left(\xi \right){\bar{u}}_{a}$$

(25) $$U_{\eta }\left(\xi ,\eta \right)=N_{a}\left(\xi \right)U_{\eta a}\left(\eta \right)$$

(26) $$U_{\zeta }\left(\xi ,\zeta \right)=N_{a}\left(\xi \right)U_{\zeta a}\left(\zeta \right)$$

(27) $$U_{\eta a}\left(\eta \right)=z_{\eta a}\left(\eta \right){\hat{U}}_{\eta a}$$

(28) $$U_{\zeta a}\left(\zeta \right)=z_{\zeta a}\left(\zeta \right){\hat{U}}_{\zeta a}$$

where *u* is the displacement of a generic point, $\bar{u}$ is the displacement of a point on the reference surface, and *U* is the ‘beam element displacement’ rotations. The motion of the beam elements can be interpreted as either displacements or rotations as will be discussed. To describe the current deformed configuration with respect to the reference configuration as schematically is shown in Figure 4b and written as follows:

(29) $$y=\bar{y}+Y\;\bar{y}=\bar{x}+\bar{u}\;{\bar{y}}_{a}={\bar{x}}_{a}+{\bar{u}}_{a}\;Y=X+U\;Y_{a}=X_{a}+U_{a}\;{\hat{Y}}_{\eta a}={\hat{X}}_{\eta a}+{\hat{U}}_{\eta a}\;{\hat{Y}}_{\zeta a}={\hat{X}}_{\zeta a}+{\hat{U}}_{\zeta a}$$

where *x* refers to the reference configuration, *y* to the deformed configuration, and *u* are the displacements. The notations with $\left(\bar{.}\right)$ indicates the reference surface quantities, as superior caret $\left(\hat{.}\right)$ indicates the unit vector quantities, lower case letter for the deformed displacements, and upper case letters for beam element displacements. Thus, to update the deformed configuration, two vector quantities are needed: the reference surface displacement $\bar{u}$ and the associated nodal beam element displacement *U*. The nodal beam element displacements are defined in the beam element coordinate system.

For a beam element, the displacements of the reference surface $\bar{u}$ obtained from the translational equations of motion and the rotational quantities at each node obtained from the rotational equations of motion. The linearized relationship between the incremental components $\Delta \hat{U}$ of the incremental rotations are given by:

(30) $$\left\{\begin{array}{c} \Delta {\hat{U}}_{\eta 1}\\ \Delta {\hat{U}}_{\eta 2}\\ \Delta {\hat{U}}_{\eta 3} \end{array}\right\}=\left[\begin{array}{ccc} 0 & {\hat{Y}}_{\eta 3} & -{\hat{Y}}_{\eta 2}\\ -{\hat{Y}}_{\eta 3} & 0 & {\hat{Y}}_{\eta 1}\\ {\hat{Y}}_{\eta 2} & -{\hat{Y}}_{\eta 1} & 0 \end{array}\right]\left\{\begin{array}{c} \Delta \theta_{1}\\ \Delta \theta_{2}\\ \Delta \theta_{3} \end{array}\right\}=h_{\eta }\Delta \theta$$

(31) $$\left\{\begin{array}{c} \Delta {\hat{U}}_{\zeta 1}\\ \Delta {\hat{U}}_{\zeta 2}\\ \Delta {\hat{U}}_{\zeta 3} \end{array}\right\}=\left[\begin{array}{ccc} 0 & {\hat{Y}}_{\zeta 3} & -{\hat{Y}}_{\zeta 2}\\ -{\hat{Y}}_{\zeta 3} & 0 & {\hat{Y}}_{\zeta 1}\\ {\hat{Y}}_{\zeta 2} & -{\hat{Y}}_{\zeta 1} & 0 \end{array}\right]\left\{\begin{array}{c} \Delta \theta_{1}\\ \Delta \theta_{2}\\ \Delta \theta_{3} \end{array}\right\}=h_{\zeta }\Delta \theta$$

These equations are used to transform the incremental beam element tip displacements to rotational increments in the equations of motion. The second-order accurate rotational update formulation is used to update the beam element vectors [54]:

(32) $${\hat{Y}}_{\eta i}^{n+1}=R_{ij}\left(\Delta \theta \right){\hat{Y}}_{\eta i}^{n}$$

(33) $${\hat{Y}}_{\zeta i}^{n+1}=R_{ij}\left(\Delta \theta \right){\hat{Y}}_{\zeta i}^{n}$$

Then:

(34) $$\Delta {\hat{U}}_{\eta a}={\hat{Y}}_{\eta a}^{n+1}-{\hat{Y}}_{\eta a}^{n}$$

(35) $$\Delta {\hat{U}}_{\zeta a}={\hat{Y}}_{\zeta a}^{n+1}-{\hat{Y}}_{\zeta a}^{n}$$

where

(36) $$R_{ij}\left(\Delta \theta \right)=\delta_{ij}+\frac{\left(2\delta_{ij}+\Delta S_{ik}\right)\Delta S_{ik}}{2D}$$

(37) $$\Delta S_{ij}=e_{ijk}\Delta \theta_{k}$$

(38) $$2D=2+\frac{1}{2}\left(\Delta \theta_{1}^{2}+\Delta \theta_{2}^{2}+\Delta \theta_{3}^{2}\right)$$

Herein, $\delta_{ij}$ is the Kronecker delta and $e_{ijk}$ is the permutation tensor.

In addition to the beam element coordinate system, also a local coordinate system is required to enforce the zero normal stress conditions transverse to the axis. The orthonormal basis with two directions ${\hat{e}}_{2}$ and ${\hat{e}}_{3}$ normal to the axis of the beam is constructed as follows:

(39) $${\hat{e}}_{1}=\frac{{\bar{y}}_{2}-{\bar{y}}_{1}}{\left\|{\bar{y}}_{2}-{\bar{y}}_{1}\right\|}$$

(40) $${\overset{´}{e}}_{2}=\frac{{\hat{Y}}_{\eta 1}+{\hat{Y}}_{\eta 2}}{\left\|{\hat{Y}}_{\eta 1}+{\hat{Y}}_{\eta 2}\right\|}$$

From the vector cross product of these local tangents.

(41) $${\hat{e}}_{3}={\hat{e}}_{1}\times {\overset{´}{e}}_{2}$$

Additionally, to complete this orthonormal basis, the vector

(42) $${\hat{e}}_{2}={\hat{e}}_{3}\times {\hat{e}}_{1}$$

is defined. This coordinate system rigidly rotates with the deformations of the element. The transformation of vectors from the global to the local coordinate system now can be defined in terms of the basis vectors as:

(43) $$\hat{A}=\left\{\begin{array}{c} {\hat{A}}_{x}\\ {\hat{A}}_{y}\\ {\hat{A}}_{z} \end{array}\right\}={\left[\begin{array}{ccc} e_{1x} & e_{2x} & e_{3x}\\ e_{1y} & e_{2y} & e_{3y}\\ e_{1z} & e_{2z} & e_{3z} \end{array}\right]}^{T}\left\{\begin{array}{c} A_{x}\\ A_{y}\\ A_{z} \end{array}\right\}=\left[q\right]\left\{A\right\}$$

where $e_{ix}$, $e_{iy}$, $e_{iz}$ are the global components of the local coordinate unit vectors, $\hat{A}$ is a vector in the local coordinates, and A is the same vector in the global coordinate system.

The next step is to calculate the incremental strain and spin tensors. These were calculated from the incremental displacement gradient as follows:

(44) $$G_{ij}=\frac{\partial ∆u_{i}}{\partial y_{j}}$$

where $∆u_{i}$ are the incremental displacements and $y_{j}$ are the deformed coordinates. The incremental strain and spin tensors are defined as the symmetric and skew-symmetric parts, respectively, of $G_{ij}$:

(45) $$∆\varepsilon_{ij}=\frac{1}{2}\left(G_{ij}+G_{ji}\right)$$

(46) $$∆\omega_{ij}=\frac{1}{2}\left(G_{ij}-G_{ji}\right)$$

The incremental spin tensor $∆\omega_{ij}$ is used as an approximation to the rotational contribution of the Jaumann rate of the stress tensor; in an implicit implementation [54]. The Jaumann rate update is approximated as:

(47) $${\underline{\sigma }}_{ij}=\sigma_{ij}^{n}+\sigma_{ip}^{n}∆\omega_{pj}+\sigma_{jp}^{n}∆\omega_{pi}$$

where the superscripts on the stress tensor refer to the updated (*n* + 1) and reference (*n*) configurations. This update of the stress is applied before the constitutive evaluation, and the stress and strain are stored in the global coordinate system.

To evaluate the constitutive relation, the stress and strain increments are rotated from the global to the local coordinate system using the transformation defined in Equation (44):

(48) $$\sigma_{ij}^{1^{n}}=q_{ik}{\underline{\sigma }}_{kn}q_{ik}$$

(49) $$\Delta \varepsilon_{ij}^{1}=q_{ik}\Delta \varepsilon_{kn}q_{jn}$$

where the superscript 1 indicates components in the local coordinate system. The stress is updated incrementally:

(50) $$\sigma_{ij}^{1^{n+1}}=\sigma_{ij}^{1^{n}}+\Delta \sigma_{ij}^{1^{n+\frac{1}{2}}}$$

and rotated back to the global system:

(51) $$\sigma_{ij}^{n+1}=q_{ki}\sigma_{kn}^{1^{n+1}}q_{nj}$$

before computing the internal force vector. After the constitutive evaluation is completed, the fully updated stresses are rotated back to the global coordinate system. These global stresses are then used to update the internal force vector as follows:

(52) $$f_{a}^{int}=\int^{\;}B_{a}^{T}\sigma d\upsilon$$

where $f_{a}^{int}$ are the internal forces at node *a* and *B_a_* is the strain-displacement matrix in the global coordinate system associated with the displacements at node *a*. The *B* matrix relates six global strain components to eighteen incremental displacements, including three translational displacements per node and the six incremental beam element tip displacements of Equations (34) and (35). It is easier to partition the *B* matrix:

(53) $$B=\left[\;B_{1},B_{2}\right]$$

Each *B_a_* submatrix is further partitioned into a portion due to strain and spin with the following submatrix definitions:

(54) $$B_{a}=\left[\begin{array}{ccccccccc} B_{1} & 0 & 0 & B_{4} & 0 & 0 & B_{7} & 0 & 0\\ 0 & B_{2} & 0 & 0 & B_{5} & 0 & 0 & B_{8} & 0\\ 0 & 0 & B_{3} & 0 & 0 & B_{6} & 0 & 0 & B_{9}\\ B_{2} & B_{1} & 0 & B_{5} & B_{4} & 0 & B_{8} & B_{7} & 0\\ 0 & B_{3} & B_{2} & 0 & B_{6} & B_{5} & 0 & B_{9} & B_{8}\\ B_{3} & 0 & B_{1} & B_{6} & 0 & B_{4} & B_{9} & 0 & B_{7}\\ & 0 & & & 0 & & & 0 & \end{array}\right]$$

where

(55) $$B_{i}=\{\begin{array}{cc} N_{a,i}=\frac{\partial N_{a}}{\partial y_{i}} & for\;i=1,2,3\\ {\left(N_{a}z_{\eta a}\right)}_{,i-3}=\frac{\partial \left(N_{a}z_{\eta a}\right)}{\partial y_{i-3}} & for\;i=4,5,6\\ {\left(N_{a}z_{\zeta a}\right)}_{,i-6}=\frac{\partial \left(N_{a}z_{\zeta a}\right)}{\partial y_{i-6}} & for\;i=7,8,9 \end{array}$$

with respect to the strain-displacement relations, the derivative of the shape functions are taken with respect to the global coordinates. In addition, the *B* matrix is computed on the cross-section located at the mid-point of the axis and the resulting *B* matrix is a 6 × 18 matrix.

The internal force, *f*, is given by:

(56) $$f^{\prime }=T^{t}f_{a}^{int}$$

is assembled into the global right-hand side internal force vector. *T* is defined as:

(57) $$T=\left[\begin{array}{cc} I & 0\\ 0 & h_{\eta }\\ 0 & h_{\zeta } \end{array}\right]$$

where *I* is a 3 × 3 identity matrix.

The integration of Equation (52) for the beam element with a tubular cross-section is performed with one-point integration along the axis and multiple points in the cross-section.

The viscoelastic material model for the beam and solid elements were defined through a shear relaxation behavior as proposed by Hermann [90]:

(58) $$G\left(t\right)=G_{\infty }+\left(G_{0}-G_{\infty }\right)\exp\left(-\beta t\right)$$

where $G_{0}$ and $G_{\infty }$ are the short-time and long-time shear moduli, respectively. The β is the decay constant.

A Jaumann rate formulation is used as follows:

(59) $$\begin{array}{c} \nabla \\ \overset{´}{\sigma_{ij}} \end{array}=2\int_{0}^{t}G\left(t-\tau \right)\overset{´}{D_{ij}}\left(\tau \right)d\tau$$

where the prime denotes the deviatoric part of the stress rate, $\begin{array}{c} \nabla \\ \overset{´}{\sigma_{ij}} \end{array}$, and the strain rate, $\overset{´}{D_{ij}}$.

### 2.3. Trabecular Meshwork Specimen Finite Element Model—Viscoelastic Parameters Calculations

**Figure 5:**
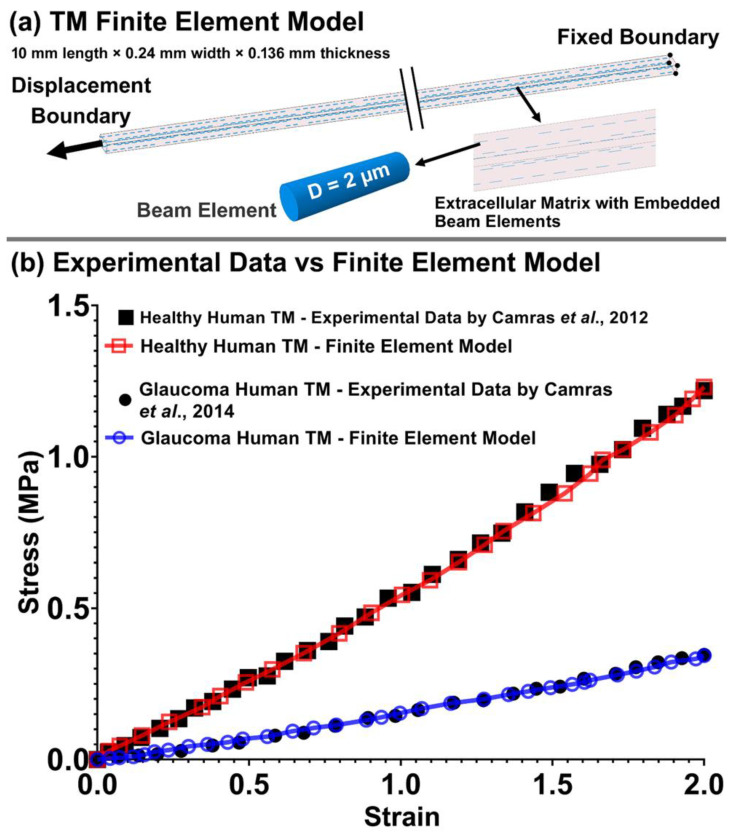
The (**a**) TM finite element model and (**b**) the experimental healthy and glaucoma human data versus the finite element models [91,92].

While there is a wide range of elastic moduli for the human TM in the literature (0.004 to 51.5 MPa [54]), we are not aware of any studies that have reported the viscoelastic mechanical properties of the TM, JCT, and SC inner wall. Thus, we pre-estimated these properties using an FE model of an experimental TM specimen. The resultant stresses were compared to the published experimental data on the healthy [91] and glaucoma [92] human TM tested in tension. The FE model of the TM specimen of 10 mm length × 0.24 mm width × 0.136 mm thickness matches the specimen dimensions in the experimental study [91,92] as shown in Figure 5a. Beam elements representing the anisotropic collagen fibrils in the TM tissue were incorporated into the specimen FE model as described in Section 2.2 and coupled to the ECM using a mesh-free, beam-in-solid algorithm. The TM specimen FE model was subjected to a uniaxial tensile strain, where the displacement boundary condition (2% strain) was applied to the FE model to mimic the uniaxial mechanical testing protocol [91,92]. The Fminsearch-Unconstrained nonlinear minimization optimization algorithm was coupled with the LS-DYNA solver to calculate the viscoelastic parameters for the ECM with embedded viscoelastic beam elements [69,93,94]. Fminsearch started with initial estimations of *G*_0_ (short-time shear modulus) = 24.5 MPa, *G*∞ (long-time shear modulus) = 17.02 MPa, and *β* (decay constant) = 500 1/s for the ECM with embedded beam elements of a healthy human eye [69]. For the glaucoma eyes, the initial estimations were *G*_0_ = 6.90 MPa, *G*∞ = 4.85 MPa, and *β* = 510 1/s for the ECM with embedded beam elements [69]. The upper and lower parameter boundaries for both the healthy and glaucoma ECM/beam elements were chosen as 0.10 < *G*_0_ < 100 MPa, 0.10 < *G*∞ < 100 MPa, and 1 < *β* <1000 1/s, based on the optimized ECM/cable elements’ mechanical properties reported in our prior publication [69]. The model was run in Matlab with the cost function of mean squared error [95] that is the sum of the squared differences between the experimental data [91,92] and optimized value. The resultant stress–strain in the gauge at the center of the TM specimen FE model (Figure 5a) was calculated and plotted versus the experimental data [91,92] as presented in Figure 5b. The optimized viscoelastic properties for the ECM and beam elements in the healthy and glaucoma FE models are summarized in Table 1.

### 2.4. TM Segmentation and Volume Meshing, TM, JCT, and SC Inner Wall Viscoelastic Parameters, and Beam Element Distribution

**Figure 6:**
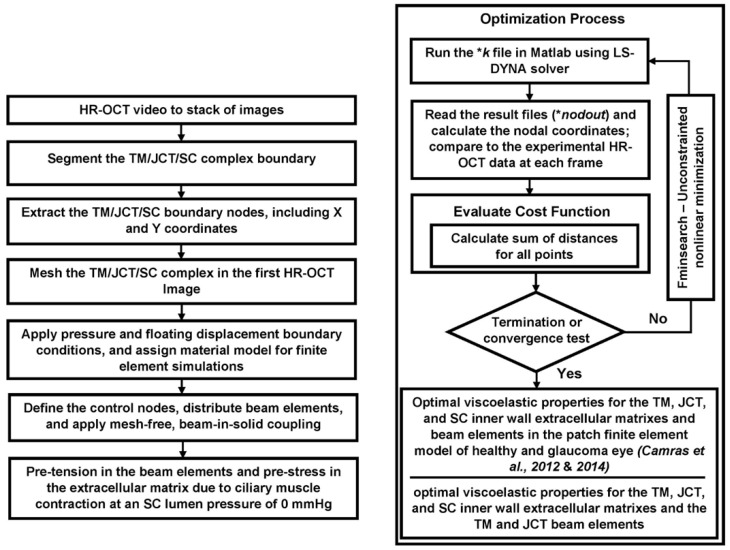
The flow-chart of the inverse finite element and optimization methods [91,92].

The flow-chart of the HR-OCT imaging, the TM/JCT/SC complex segmentation, nodal coordinates extraction, and optimization process to calculate the viscoelastic mechanical properties of the ECM/beam elements is shown in Figure 6.

**Figure 7:**
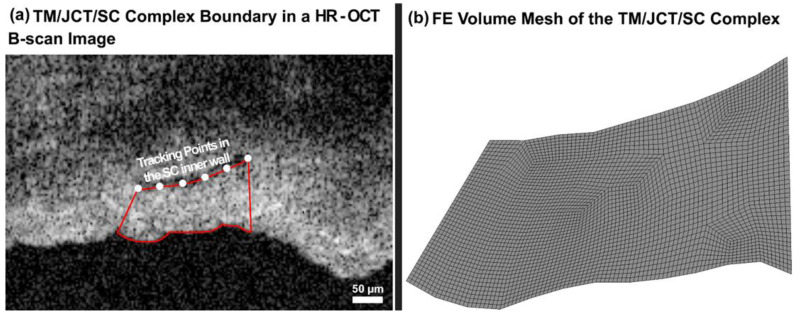
The (**a**) TM/JCT/SC complex boundary in the HR-OCT B-scan Image. (**b**) Finite element mesh of the TM/JCT/SC complex. The dynamic motion of defined points in the SC inner wall were tracked throughout the pressure elevation.

The HR-OCT with 30 B-scans/second at an appropriate distance from the cannula provided a set of dynamic images of the TM/JCT/SC complex as the pressure in the SC elevates from 0 to 30 mmHg. The TM/JCT/SC complex delineation and volume meshing are fully explained in our prior publication [69]. Briefly, HR-OCT video data were converted to a stack of images and delineated [94] under the supervision of an expert glaucoma specialist (MJ) as shown in Figure 7a. The nodal coordinates in the boundaries of the TM/JCT/SC complex were used to define the floating displacement boundary condition for the following FE simulations.

To eliminate the sharp vertices in the FE mesh, the boundaries of the TM/JCT/SC complex were smoothed in the HR-OCT images as described in our prior publication [69] using a smoothing spline algorithm [96]:

(60) $$Smoothing\;Algorith\;\left(SA\right)=p\underset{i}{\sum }w_{i}{\left(y_{i}-s\left(x_{i}\right)\right)}^{2}+\left(1-p\right)\int^{\;}{\left(\frac{d^{2}s}{dx^{2}}\right)}^{2}dx$$

where the smoothing spline s is made for the specified smoothing parameter *p* = 0.999 and the specified weights wi [69]. The first 2D HR-OCT images were delineated as described above, extruded to 10-µm thickness, and volume meshed [83] using an 8-noded hexahedral element type with fully integrated element formulation as shown in Figure 7b.

**Figure 8:**
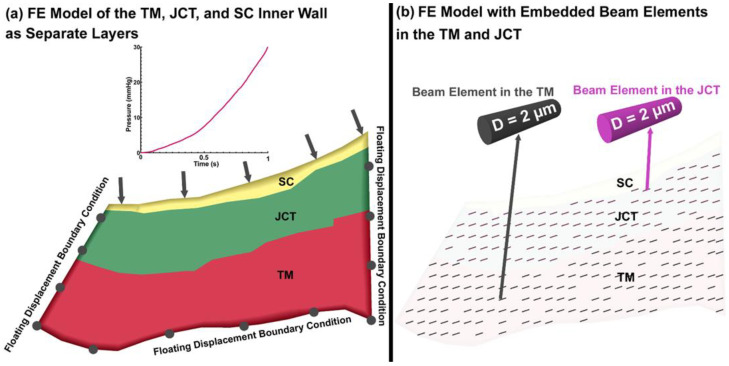
(**a**) The finite element model of the TM, JCT, and SC inner wall as separate layers. (**b**) The finite element model of the TM/JCT/SC complex with embedded beam elements.

The FE mesh was separated into the TM, with adjacent JCT and SC inner wall regions with thicknesses of ~14 μm [97] and ~2.2 μm [47], respectively, as shown in Figure 8a. The control points were distributed into the outflow tissues’ solid matrix (surface mesh *STL) using a custom Matlab program for further beam element distribution [68]. The distance between the control points of the beam elements was set to 4 µm (planar) and 2.5 µm (through the thickness), which is restricted by the 10-µm thickness of the model. The beam elements were distributed in an asymmetric fan-shaped configuration parallel to the external and internal edges of the TM [17,80,98,99,100,101,102,103,104] using a custom Matlab program as shown in Figure 8b. The nodal coordinates (X, Y) in the TM/JCT/SC complex from the HR-OCT imaging data were calculated and applied to the model as a floating displacement boundary condition as explained in our prior publications [69,94]. The pressure boundary was applied in the SC inner wall based on the optimized pressure profile reported in our prior publication [69]. The solid TM, JCT, and SC inner wall solid matrixes were modeled as a viscoelastic material using 8-noded hexahedral elements with Galerkin element-free formulation [105,106].

**Figure 9:**
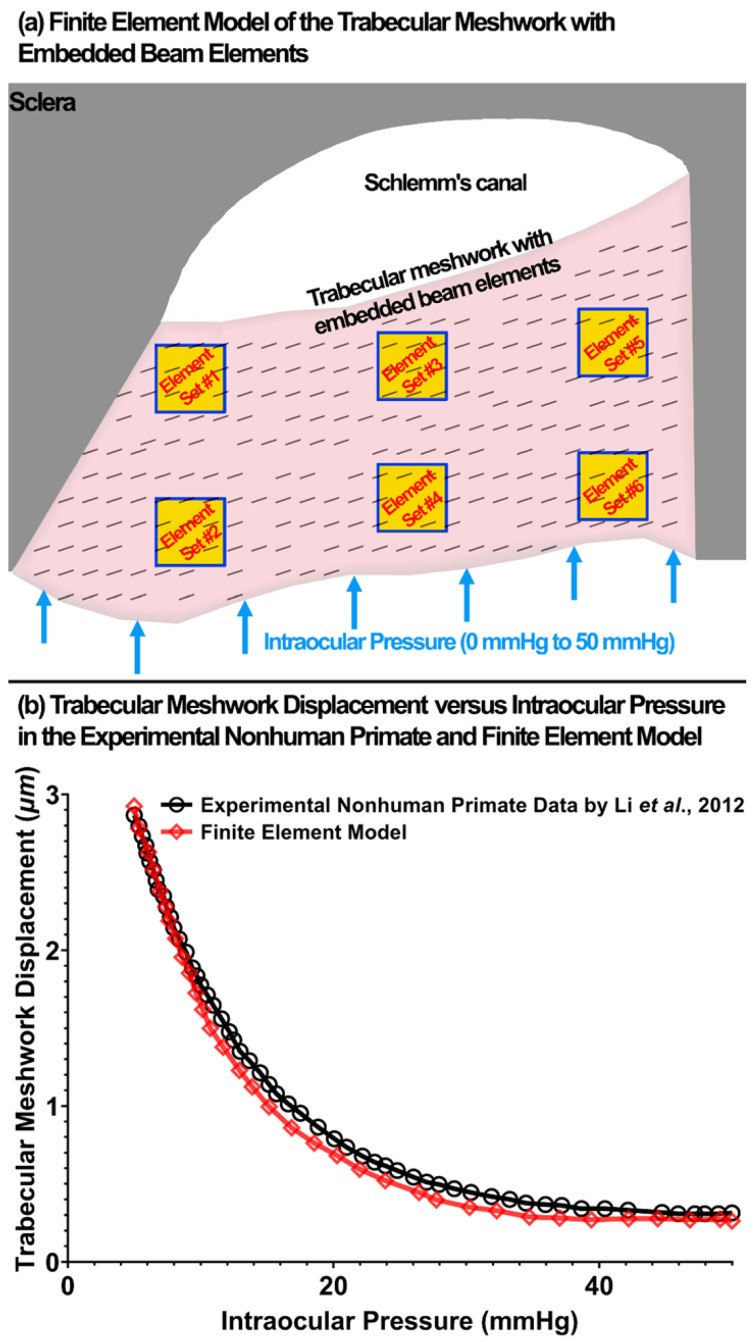
(**a**) The finite element model of the TM with embedded beam elements. Six different element sets were defined in the model and the model was subjected to IOP elevation. (**b**) TM displacement versus the IOP in the experimental nonhuman primate [53] and finite element model.

The term *β* (decay constant) in a stress relaxation function affects the rise and relaxation mechanical response of a viscoelastic tissue. However, since rising usually happens in a shorter but the relaxation in a longer time, it is more accurate to calculate the β considering both the rising and relaxation response of a tissue. The available uniaxial tensile test for a healthy human TM [91] does not include any time-dependent mechanical response of the tissue. However, Li [36] reported the displacement in the TM as a function of IOP elevation from 5 to 50 mmHg in nonhuman primates (*Macaca nemestrina*), so the time-dependent mechanical response of the tissue is already included in the TM displacement. The β was calculated from the IOP-TM displacement data, and then it was considered as a constant parameter for the rising part. To do that, the FE model of the TM/JCT/SC complex was used and subjected to IOP elevation from 0 to 50 mmHg in 1 s (Figure 9a). The elastic modulus of the sclera was 2.93 MPa [107] and nearly incompressible (Poisson’s Ratio, *ν* = 0.495) [108,109]. The Fminsearch-Unconstrained nonlinear minimization optimization algorithm was coupled with the LS-DYNA solver to calculate the viscoelastic parameters for the ECM of the TM with embedded beam elements. The initial estimations were *G*_0_ = 24.98 MPa, *G*∞ = 18.81 MPa, and *β* = 500 1/s for the ECM (Table 1). In the beam elements, the initial estimations were *G*_0_ = 35.2 MPa, *G*∞ = 20.51 MPa, and *β* = 585 1/s (Table 1). The upper and lower parameter boundaries for the ECM/beam elements were 0.10 < *G*_0_ <100 MPa, 0.10 < *G*∞ < 100 MPa, and 1 < *β* < 1000 1/s. Six node sets were randomly selected in the TM FE model and the average displacement in those nodes were calculated after each optimization iteration (Figure 9a). The model was run in Matlab with the cost function of mean squared error between the TM displacement data [36] and average nodal displacement in the TM FE model. The TM displacement versus the IOP is plotted in Figure 9b. The optimized viscoelastic parameters for the healthy TM patch FE model are listed in Table 2.

The *β* for the TM, JCT, SC inner wall, and the beam elements in the TM and JCT were assumed to be constant and equal to 109 1/s and 450 1/s for the ECM and beam elements, respectively (Table 2). The initial estimations were then changed to *G*_0_ = 24.98 MPa, *G*_∞_ = 18.81 MPa, and *β* = 109 1/s for the ECM of the TM, JCT, and SC inner wall (Table 1 and Table 2). In the beam elements, the initial estimations were *G*_0_ = 35.2 MPa, *G*_∞_ = 20.51 MPa, and *β* = 450 1/s (Table 1 and Table 2). The upper and lower parameter boundaries for the ECM/beam elements were chosen as 0.10 < *G*_0_ < 100 MPa and 0.10 < *G*∞ < 100 MPa. The model was run with the cost function of mean squared error between the SC inner wall nodal coordinates of the HR-OCT experimental data and optimization data. The optimized viscoelastic properties for the TM, JCT, and SC inner wall, and beam elements are reported in Table 3 and Table 4.

### 2.5. Parameter Uniqueness in Fminsearch-Unconstrained Nonlinear Minimization

While the optimized material parameters (Table 3 and Table 4) may not be an absolute minimum for a non-convex cost function, they are the best “possible” minimum solution after more than ~100 iterations. Fminsearch-Unconstrained nonlinear minimization finds the minimum of an unconstrained multivariable function using the derivative-free method [110]. Fminsearch is a multivariate curve resolution (MCR) method such that uncertainty in the parameters due to a wide range of initial guesses may invoke non-unique results [111]. The non-uniqueness problem is an inevitable part of MCR methods. However, an intelligent selection of data structure and provision of reasonable constraints may significantly alleviate or even eliminate the non-uniqueness problem in some cases [112,113]. Here, the parameters were constrained with suitable upper and lower bounds [69], which helped to eliminate any observably non-physiologic results. To check the reliability or uniqueness of the results, we perturbed the system through choosing different initial guesses [110,113]. Thus, eight different sets of initial guesses, defined as 10%, 20%, 30%, and 40% greater and lesser than the optimized material parameters, were assigned to a healthy and glaucoma TM/JCT/SC. The complex FE model and the converged properties were compared to those of the optimized set of parameters (Table 3 and Table 4). In all cases, optimization resulted in similar parameters (less than ~10% difference), so we can claim that the solution is consistent but not a relative minimum.

### 2.6. Element Quality Assessment and Continuum Wave Propagation Velocity in 3D Elements

**Figure 10:**
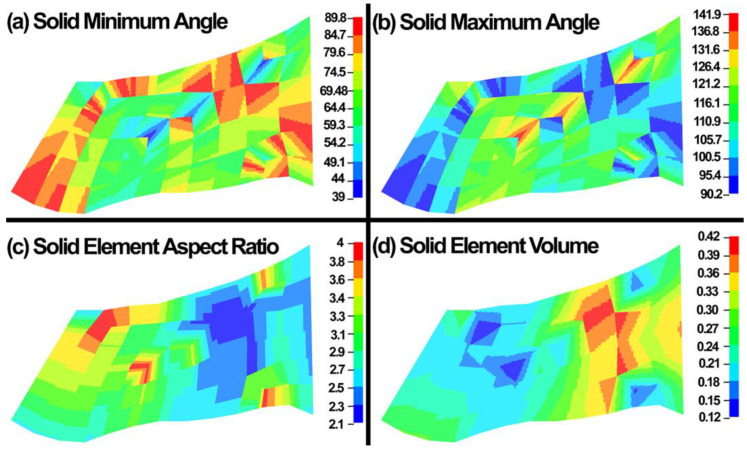
(**a**) Solid minimum angle, (**b**) solid maximum angle, (**c**) solid element aspect ratio, and (**d**) solid element volume (µm^3^) in the FE model.

The element quality assessment was carried out for the FE models to make sure the resultant meshes are well within the range of acceptable elements. The element minimum and maximum angles, element aspect ratio, and element volume were calculated and shown in Figure 10. The element volume shows that it is important to report volumetric average stresses or strains in the FE models since the distribution of the element volume in a FE model may significantly affect the stress or strain map in a model.

Time step size is the minimum division of the time on which the maximum iteration you have given is going to perform. In explicit dynamic simulations, the time step plays an important role to secure a stable solution. Explicit time integration scheme is conditionally stable and the global computing time step must be less than models highest natural frequency that in the LS-DYNA it should be <10% of the reduction factor. In the LS-DYNA, the highest natural frequency is approximated as the ratio of the characteristic length and the sound speed [114]. In LS-DYNA, it is defined through the dilation wave in a solid element that is calculated as follows:

(61) $$C=\sqrt{\frac{E\left(1-\nu \right)}{\left(1+\nu \right)\left(1-2\nu \right)\rho }}$$

where the E is the Young’s modulus, ν is the Poisson’s ratio, and ρ is the specific mass density. The critical time step for the dynamic simulation is then calculated as follows:

(62) $$∆t_{min}=\frac{l}{C}$$

where l is the length of the element [114]. The l for an 8-noded hexahedral element is as follows:

(63) $$∆t_{min}=\frac{V_{e}}{A_{e-max}}$$

where $V_{e}$ is the element volume and $A_{e-max}$ is the area of the largest side, and c is the plane stress sound speed:

(64) $$c=\sqrt{\frac{E}{\rho \left(1-\nu^{2}\right)}}$$

**Figure 11:**
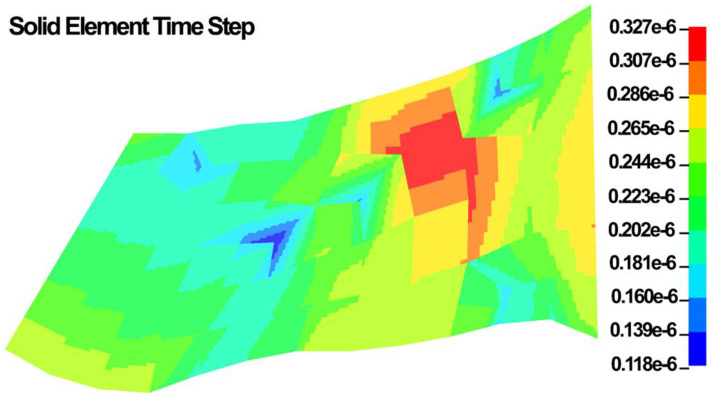
Solid element type step in the FE model.

In LS-DYNA, the time step size should not exceed 0.456 × 10^−6^ to avoid contact instabilities to assure stable results. The contour maps of the time step in the FE model is shown in Figure 11.

### 2.7. Statistical Analysis

Data from the simulation of four eye-specific FE models were determined to be normally distributed. The statistical significance of the difference between sample means was evaluated using a randomized one-way analysis of variance (ANOVA). When indicated by a significant F statistic after a one-way ANOVA, post hoc comparisons with the Scheffe method [115] were used to determine the individual levels of significant differences among the material parameters for the ECM and beam elements. The criterion chosen to discard the null hypothesis was *p* < 0.05.

## 3. Results

**Figure 12:**
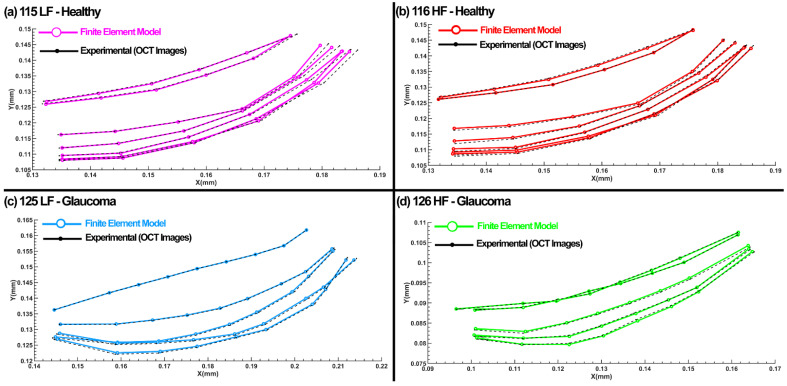
Nodal coordinates of the SC inner wall with SC pressure elevation in both the FE model and experimental HR-OCT imaging data in (**a**) 115LF—Healthy, (**b**) 116HF—Healthy, (**c**) 125LF—glaucoma, and (**d**) 126HF—glaucoma eyes.

The diameter of each cross-section in the SC at different distances from the cannula (Figure 3g) was measured through a Matlab code. The average diameter in each of the four cross-sections was 126 ± 5 µm within 800 µm from the cannula, which assures us that the pressure in these regions can be assumed to be equal immediately distal to the cannula. The best-optimized shape of the SC inner wall that resulted in the best possible match with the TM/JCT/SC complex FE model and the experimental HR-OCT data are shown in Figure 12. The optimized viscoelastic parameters for the healthy TM, JCT, and SC inner wall with embedded beam elements are shown in Table 3 and Table 4.

## 4. Discussion

It is known that soft biological tissues [62,63,64,65,66,67], especially the TM [36,58], are anisotropic and viscoelastic. Characterizing the time-dependent mechanical behavior of the outflow tissues with a dynamic load boundary may contribute to our understanding of active IOP regulation in the human eye [17]. Experimental [116,117,118,119,120,121,122], numerical [123,124,125], and review [9,10,17,126,127,128,129,130,131,132] studies have all tremendously contributed to a better understanding of the mechanism of outflow resistance in the conventional outflow pathway. However, the active outflow tissues’ biomechanical responses are largely unknown. The outflow pathway is a very dynamic mechanical environment that actively affects the tissues’ geometry [120,133] through a coupling between outflow hydrodynamics and the TM, JCT, and SC inner wall that form a fluid–structure interaction [28].

In this study, one end of SC in a quadrant of the anterior segment in LF and HF segmental regions of two human donors were cannulated, and pressures switched from 0 to 30 mmHg (Figure 1, Figure 2 and Figure 3). Hariri using a precise perfusion pump measured the area in the SC as a function of the pressure, and reported the SC diameter of 124.66 ± 11.5 mmHg [80], that is in a very good agreement with our results (126 ± 5 µm). This assures us the pressure of the SC in our FE simulations can be assumed to be ~30 mmHg. A series of HR-OCT cross-sectional B-scans captured the TM/JCT/SC complex motion during SC pressurization. An inverse FE method coupled with the optimization algorithm was used to calculate the mechanical properties of the TM, JCT, and SC inner wall with embedded beam elements (Figure 4, Figure 5, Figure 6, Figure 7, Figure 8, Figure 9, Figure 10 and Figure 11 and Table 1, Table 2, Table 3 and Table 4). The inverse FE method coupled with the optimization algorithm resulted in the best possible match between the SC nodal coordinates of the HR-OCT imaging data and that of the FE models during SC pressure elevation (Figure 12).

Vranka using AFM showed that the elastic modulus of the TM in the LF regions at the pressure of 8.8 mmHg is 14.98 kPa while in the HF region is 6.49 kPa. When they elevated the pressure to 17.6 mmHg, the elastic modulus of the TM in the LF region is 30.33 kPa while in the HF region is 2.72 kPa [58,134]. Keller similarly showed that the HF regions have a lower elastic modulus and are more compliant than the LF regions [135]. Our results are in very good agreement with Vranka and Keller, as we found larger short- and long-time shear moduli for the LF regions compared to the HF regions (Table 3 and Table 4), so the HF regions are more compliant and dynamic in their homeostatic response to elevated pressure. In both the LF and HF regions, the TM had the largest while the JCT had the smallest shear moduli. Larger shear moduli in the TM compared to the JCT and SC inner wall may relate to its central role in maintaining aqueous outflow resistance [58]. The beam elements in the LF regions of the TM and JCT showed larger shear moduli compared to the HF regions, with larger shear moduli also present for the beam elements in the TM region compared to the JCT (Table 3 and Table 4).

Collagen stiffness has been indicated to play a crucial role in IOP elevation of the outflow pathway [136,137]. The collagen fibrils in the TM lamellae primarily controls the TM distension and recovery to IOP fluctuation [138]. Our results showed significantly larger shear moduli in the ECM of the glaucoma eyes compared to the healthy eyes (*p* = 0.005, Table 3 and Table 4) that are in good agreement with findings on the TM stiffness in glaucoma eyes [10,12,14,30,32,51,54,59,61,139,140]. Moreover, it has been shown that the ECM of the glaucomatous TM loses its resiliency and becomes stiffer [141], so it is reasonable to speculate that the collagen fibrils in the glaucoma eyes are stiffer than in healthy eyes [54,142]. This result is in good agreement with our findings (Table 3 and Table 4), as the beam elements in the glaucoma eyes showed significantly larger short-time (TM = 2.4-fold, JCT = 1.9-fold) and long-time shear moduli (TM = 2-fold, JCT = 1.8-fold) compared to the healthy eyes (*p* = 0.001).

Aging or chronic exposure to ocular hypertension may result in elastin fragmentation and replacement by collagen that causes a stiffer mechanical response in the TM of the glaucoma eyes [54,142,143]. Large deformation in the TM due to higher IOPs produces more collagen that reduces the elastin: collagen ratio and stiffens the TM [144,145,146]. Our results revealed 2.4 and 1.9-fold larger short-time shear moduli in the beam elements of the TM and JCT in the glaucoma eyes compared to the healthy eyes (Table 3 and Table 4). The long-time shear moduli of the beam elements were also 2 and 1.8-fold larger in the TM and JCT of the glaucoma eyes compared to the healthy eyes (Table 3 and Table 4). Our recent study also showed 1.82 stiffer cable element elastic moduli in the glaucoma eyes compared to the healthy eyes [69] which is in good agreement with the current findings. Based on these results, it is reasonable to speculate that the stiffer response of the beam elements in the glaucoma eyes implies the presence of additional collagen fibrils in the ECM of the TM and JCT of glaucoma eyes [58,147] or additional cross linking [148].

The IOP elevation from 8 to 30 mmHg could cause up to 50% stretch in the cells of the outflow pathway [149]. Thus, the effects of mechanical stress in the SC cell basement membrane ECM may be considered as one potential mechanism to counteract pressure fluctuations and regulate the outflow facility [150]. The viscoelastic outflow tissues actively respond to pressure fluctuation, providing a means to better address the tissues’ mechanical response with an IOP elevation. In addition to mechanical strain associated with elevating IOP, the TM experiences smaller pulsatile distensions associated with the ocular pulsations [14]. It has been shown that with developing glaucoma, pulsatile aqueous humor flow diminishes until it is eventually absent [15,37].

### Limitations

First, the SC is pressurized in our ex vivo preparation, although the aqueous humor applies pressure on the TM in vivo; hence, our experimental setup is the reverse of the in vivo condition. In addition, the SC lumen is pressurized to 30 mmHg in our experiments, while prior work has shown that the physiological pressure in the SC is 5.6–10.5 mmHg [26,151]. However, the large deformations in the outflow tissues resulting from this approach allowed us to calculate the viscoelastic properties of the TM, JCT, and SC inner wall as separate tissues, which would be difficult to achieve with physiologic pressures applied to the TM from inside the eye. In our future organ culture perfusion setup, we aim to use different pressure ranges, such as 0–8.8, 0–17.6, and 0–30 mmHg, to have wide range of small and large deformations in the tissues. In addition, the ocular pulse amplitude has not been included in the experimental setup. While this can be considered as a limitation of the experimental setup, the ocular pulse amplitude only alters the load boundary and would not affect the resultant mechanical properties of the outflow tissues. In this study, the mechanical properties of the outflow tissues were calculated with dynamic SC pressurization from 0 to 30 mmHg using finite element method-optimization algorithm based on the high-resolution OCT images.

Second, the TM boundary was segmented and the JCT and SC inner wall were separated from the TM FE model based on the average tissue thicknesses available in the literature. Although the current imaging technique provided us with a suitable resolution to determine the TM boundary, it is not high enough to accurately determine the boundaries between the TM, JCT, and SC inner wall. Imaging of a tissue in situ with a dynamic load boundary is very challenging, and HR-OCT is one of the best available imaging approaches for the task.

Third, one may argue that Camras et al. [91,92] data are outside the physiologic range based on measurements using other methods. However, it should be noted that these data were only used to train TM FE model in the first step of the analysis. After this initial guess based on the Camras data, another cycle of the FE-optimization algorithm was used for parameter selection in a manner that is completely independent from the Camras data. Thus, it is unlikely that the resultant optimized viscoelastic material properties were affected by the Camras data. Further, the optimized results were perturbed to ensure the properties are fully independent of the input data. In addition, prior studies have reported tensile moduli of the TM in human and porcine eyes on the order of megapascals (MPa), while others using the AFM and FEM reported the TM moduli on the order of kilopascals (kPa). The TM and JCT are collagenous tissues, so only tensile biomechanical measurements account for the important mechanical roles of the collagen and elastin fibrils. AFM is local indentation (compression) and so this technique ignores the fibrils’ biomechanical role. Hence, the strengths and weaknesses inherent in the testing methods need to be considered.

Fourth, the length, diameter, stiffness, and distribution of the beam elements representing the anisotropic stiffness imparted by collagen fibrils were assumed to be uniform throughout the entire TM and JCT, even though collagen fibrils have been shown to be thicker closer to the anterior chamber [98]. However, the quantitative data on collagen fibril characteristics in the TM and JCT is sparse, so we will incorporate heterogeneous beam element distributions in our future models as those data become available. In the current models, the beam elements were distributed according to the literature, and the density of the beam elements was constrained to the geometry of the TM and JCT.

Finally, we only used four samples from each of two healthy and two glaucoma eyes. While we did find statistically significant differences in our results, future studies will benefit from a larger cohort of healthy and glaucoma eyes, considering the age, race, sex, and disease severity of the human donors. In addition, patients with glaucoma were likely under treatment with pressure-lowering medications, which affect both IOP and TM/JCT/SC biomechanics. In addition, we only studied samples from the temporal quadrant, and future study will be necessary to determine the mechanical properties around the entire circumference of the outflow pathway.

## 5. Conclusions

In this study, the SC was pressurized in an oscillating manner from 0 to 30 mmHg in wedges of healthy and glaucoma human donor eyes, and the outflow pathway HR-OCT imaged. The inverse FE-optimization algorithm was used to calculate the viscoelastic mechanical properties of the TM, JCT, and SC inner wall with embedded viscoelastic beam elements. The finding of this study are as follows:

(a) Significantly larger time-dependent shear moduli for the ECM and beam elements in the glaucoma eyes compared to the healthy eyes.
(b) ECM and beam elements in glaucoma tissues showed larger shear moduli than the heathy tissues.
(c) TM showed larger shear moduli compared to the JCT and SC inner wall.
(d) The LF regions of the outflow tissues showed a stiffer mechanical response compared to the HF regions.
(e) Models that account for the time-dependent mechanical responses of the outflow tissues will help to improve accuracy of numerical models of the aqueous outflow system.
(f) Such models will further the study of tissue dynamics that regulate aqueous outflow. Thus, these models may provide new perspectives in understanding, diagnosing, and treating ocular hypertension and glaucoma.

## Figures and Tables

**Table 1:** Viscoelastic parameters for the healthy and glaucoma TM patch FE models. The ECM and beam elements were modeled as viscoelastic material.

Viscoelastic Parameters	*G*_0_ (MPa)	*G*_∞_ (MPa)	*β* (1/s)
Healthy Eyes			
Extracellular matrix	24.98	18.81	500
Beam Element	35.2	20.51	585
Glaucoma Eyes			
Extracellular matrix	8.15	4.45	510
Beam Element	45.88	19.58	610

**Table 2:** Viscoelastic parameters for the TM patch FE model. The ECM and beam elements were modeled as the viscoelastic material. The *β* was fixed in this cycle of optimization and the time-dependent shear moduli were optimized.

Viscoelastic Parameters	*G*_0_ (MPa)	*G*_∞_ (MPa)	*β* (1/s)
Extracellular matrix	21.19	15.98	109
Beam Element	36.19	19.58	450

**Table 3:** Viscoelastic parameters for the TM, JCT, and SC inner wall FE model in healthy eyes. The ECM and beam elements were modeled as the viscoelastic material.

Viscoelastic Parameters	*G*_0_ (MPa)	*G*_∞_ (MPa)	*β* (1/s)
115HF (Male, 54 y, postmortem time = 43 h)			
TM	3.85	3.18	109
JCT	1.15	1	109
SC	2.55	1.12	109
TM Beam Element	92.20	58.51	450
JCT Beam Element	65.20	32.51	450
115LF (Male, 54 y, postmortem time = 43 h)			
TM	5.10	4.75	109
JCT	3.15	1.49	109
SC	4.55	2.12	109
TM Beam Element	95.26	70.59	450
JCT Beam Element	75.20	39.51	450
116HF (Male, 89 y, postmortem time = 45 h)			
TM	3.05	2.26	109
JCT	1.02	0.93	109
SC	2.14	1.95	109
TM Beam Element	85.14	50.15	450
JCT Beam Element	70.16	40.25	450
116LF (Male, 89 y, postmortem time = 45 h)			
TM	4.02	2.95	109
JCT	2.35	1.55	109
SC	3.12	2.08	109
TM Beam Element	90.54	55.15	450
JCT Beam Element	75.75	45.19	450

**Table 4:** Viscoelastic parameters for the healthy TM, JCT, and SC inner wall FE model in glaucoma eyes. The ECM and beam elements were modeled as the viscoelastic material.

Viscoelastic Parameters	*G*_0_ (MPa)	*G*_∞_ (MPa)	*β* (1/s)
125HF (Female, 80 y, postmortem time = 67 h)			
TM	12.51	5.15	109
JCT	4.18	2.2	109
SC	6.28	3.18	109
TM Beam Element	205.54	109.41	450
JCT Beam Element	132.12	55.39	450
125LF (Female, 80 y, postmortem time = 67 h)			
TM	16.11	7.15	109
JCT	9.05	3.05	109
SC	12.59	4.16	109
TM Beam Element	255.15	152.28	450
JCT Beam Element	129.69	85.98	450
126HF (Female, 80 y, postmortem time = 67 h)			
TM	15.14	6.58	109
JCT	4.52	2.05	109
SC	6.38	3.88	109
TM Beam Element	198.25	98.14	450
JCT Beam Element	145.69	68.15	450
126LF (Female, 80 y, postmortem time = 67 h)			
TM	18.88	7.19	109
JCT	5.18	3.38	109
SC	7.22	4.97	109
TM Beam Element	215.44	113.34	450
JCT Beam Element	145.65	75.95	450

## Data Availability

The raw/processed data required to reproduce these findings cannot be shared at this time as the data is part of an ongoing study.
